# Metastatic Melanoma: A Consequence of the COVID-19 Pandemic

**DOI:** 10.7759/cureus.38397

**Published:** 2023-05-01

**Authors:** Robert L Phillips, Minhee P Moody, Marc H Hohman, John J Fowler

**Affiliations:** 1 Medicine, Madigan Army Medical Center, Tacoma, USA; 2 Medicine, Eisenhower Army Medical Center, Augusta, USA; 3 Otolaryngology, Madigan Army Medical Center, Tacoma, USA; 4 Pathology, Madigan Army Medical Center, Tacoma, USA

**Keywords:** skin cancer prevention, nasal reconstruction, metastatic melanoma, telehealth, covid-19, melanoma

## Abstract

Metastatic melanoma, though less common than other skin cancers, remains one of the deadliest, particularly in late-stage disease. Our report aims to highlight the importance of early detection and treatment to reduce the morbidity, mortality, and significant disfigurement associated with advanced melanoma. The subject of this case is an 81-year-old female who presented to our emergency department as a trauma patient after being found lying down by a neighbor for an unknown amount of time. She was discovered to have a large fungating nasal mass which was subsequently diagnosed as highly invasive melanoma. A thorough workup revealed a metastatic cerebellar lesion, a large ulcerated basal cell carcinoma eroding her calvarium, and a hemorrhagic lesion within her internal capsule that left her with right-sided hemiparesis. During hospitalization, she underwent palliative resection of the primary nasal mass with flap reconstruction, radiation therapy for her cerebellar lesion, and daily physical therapy. Additional surgery was required for hematoma evacuation and pedicle dissection. Though lockdowns were an important part of the pandemic, they were not without their drawbacks, many of which are still being elucidated. Particularly, by utilizing telehealth services, our patient may have had earlier recognition of her melanoma and a better outcome. Regardless, enhancing patient education and maintaining access to care even through lockdowns poses a potential target for improving melanoma survivability while decreasing associated morbidity.

## Introduction

Skin cancer, both non-melanoma and melanoma combined, continues to be one of the most commonly diagnosed cancers with roughly 1.5 million new cases and more than 120,000 deaths annually [1]. Though less common than other skin cancers, malignant melanoma remains the most deadly, with a five-year survival rate that ranges from 99% for localized disease to a mere 30% in melanoma with distant metastasis [2]. Unfortunately, data evaluating the clinical impact of the COVID-19 pandemic on the initial prognosis of malignant melanoma, as well as survival rates, is sparse. However, several studies and surveys, including the Research and Development Survey by the National Center for Health Statistics, were initiated to clarify the effect the pandemic has had on access to medical care [3]. Herein, we aim to highlight the importance of routine patient education and early disease detection to prevent significant complications in a patient whose emergent presentation was driven by pandemic-induced fear.

## Case presentation

An 81-year-old female with no known past medical history presented to our emergency department via ambulance after being found on the floor by her neighbor. Initially unresponsive, subsequent history revealed that she had been dizzy prior to a ground-level fall and was physically unable to recover until her neighbor found her. The patient stated that over the course of two years, a mass on the side of her nose had been enlarging and intermittently bleeding. However, she actively avoided contacting her physician or scheduling a medical appointment due to fears surrounding the pandemic and the possibility of contracting SARS-CoV2.

**Figure 1:**
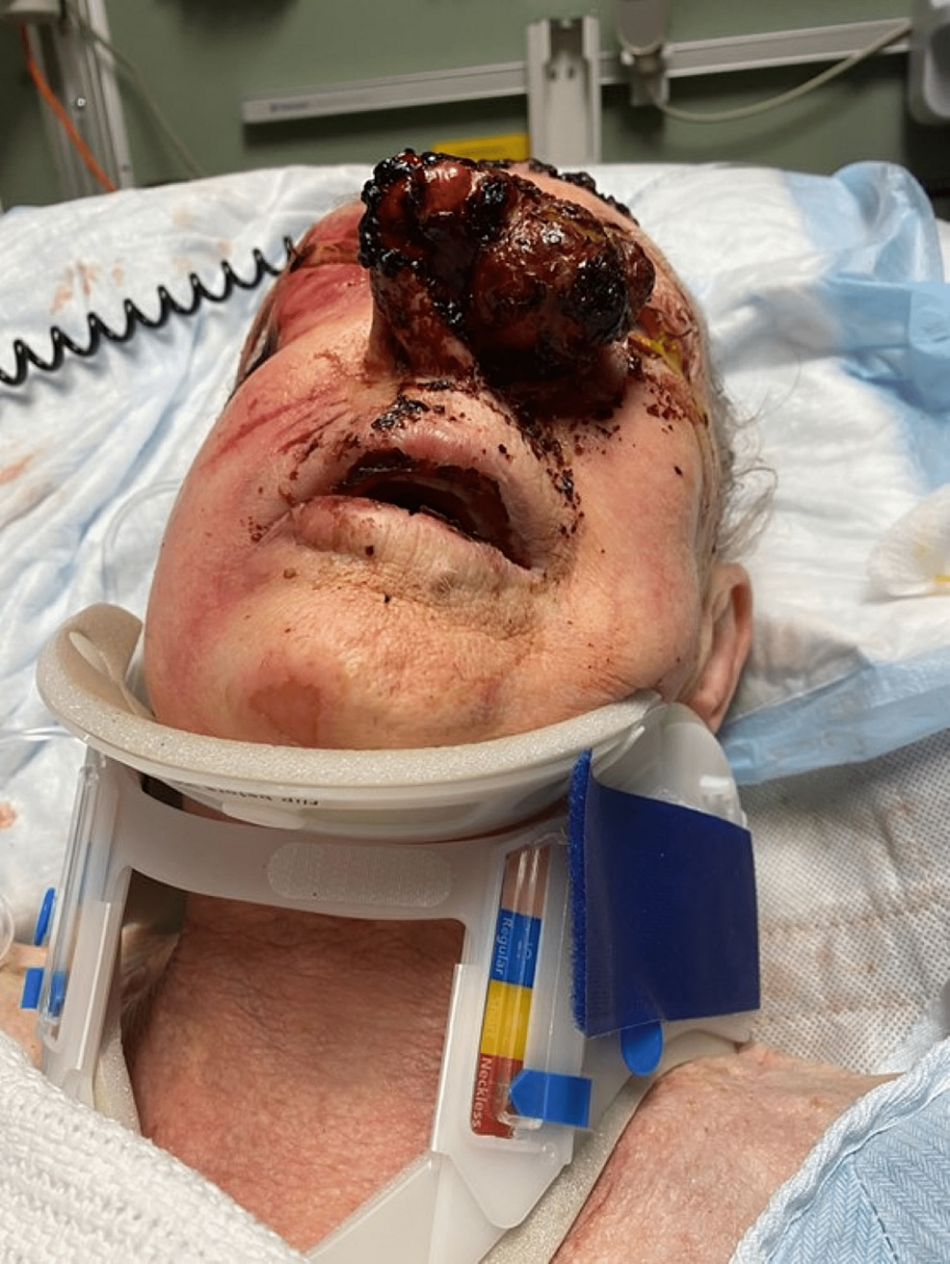
Nasal melanoma A primary survey revealed a large fungating nasal mass that was biopsied, subsequently returning as melanoma.

In the emergency department, initial primary and secondary surveys revealed several injuries including a full-thickness pressure injury to the right temple and periorbita resulting in exposed zygomatic periosteum; a dislocated left shoulder; a 7 cm x 7 cm well-circumscribed, ulcerated skin lesion with a bleeding base on the left frontoparietal scalp; and a 3 cm x 5 cm exophytic, fungating mass on the left nasal sidewall with multiple lobes and an overlying black eschar. This easily friable nasal mass involved the entirety of the left ala and sidewall, extended laterally past the nasofacial groove into the medial cheek and medially across the facial midline to cover the nasal tip, distorting the columella and right ala (Figure 1). Neurological examination revealed flaccid paralysis of the upper and lower extremities.

**Figure 2:**
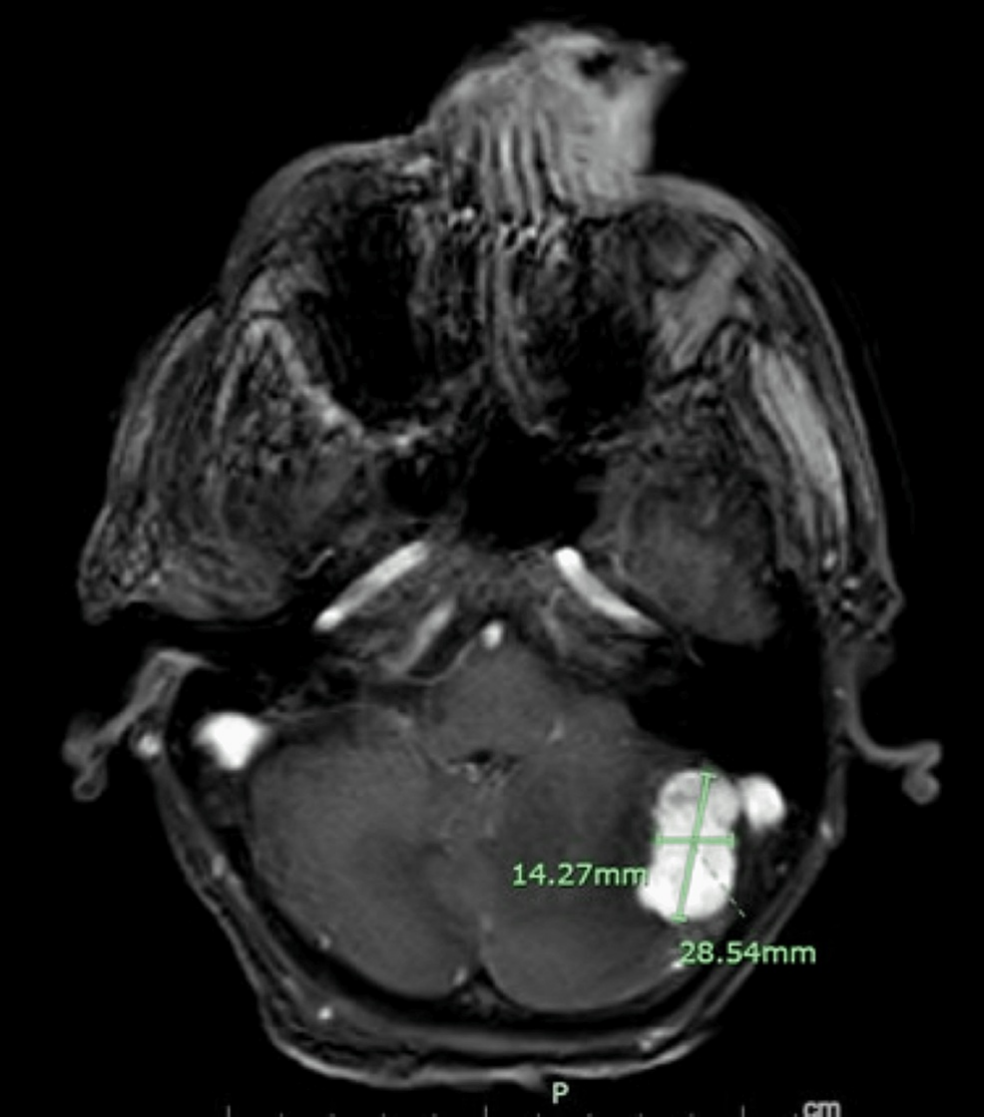
Metastatic cerebellar mass The MRI T1-weighted FFE image shows the enhancing 2.8 cm left cerebellar hemisphere mass. FFE: Fast field echo

Laboratory studies revealed an elevated serum creatinine, creatine kinase, and troponin. Electrocardiogram showed no evidence of ischemia. The nasal mass was biopsied and sent to the pathology lab for further identification. Computed tomography of the head demonstrated a 2.8 cm enhancing mass within the left lateral cerebellum. Magnetic resonance imaging of the brain showed this mass to be diffusely hypointense on T1 and hyperintense on T2, with surrounding vasogenic edema and lateral effacement of the fourth ventricle (Figure 2).

The patient was initially admitted to the intensive care unit by the trauma surgery service for hourly neurological exams in the context of polytrauma. Surgical intervention was considered after otolaryngology, neurosurgery, and ophthalmology services were consulted. Once medically stable, she was transferred to the inpatient ward for further workup of multiple masses and treatment of rhabdomyolysis. She had daily physical and occupational therapy for residual right-sided weakness.

**Figure 3:**
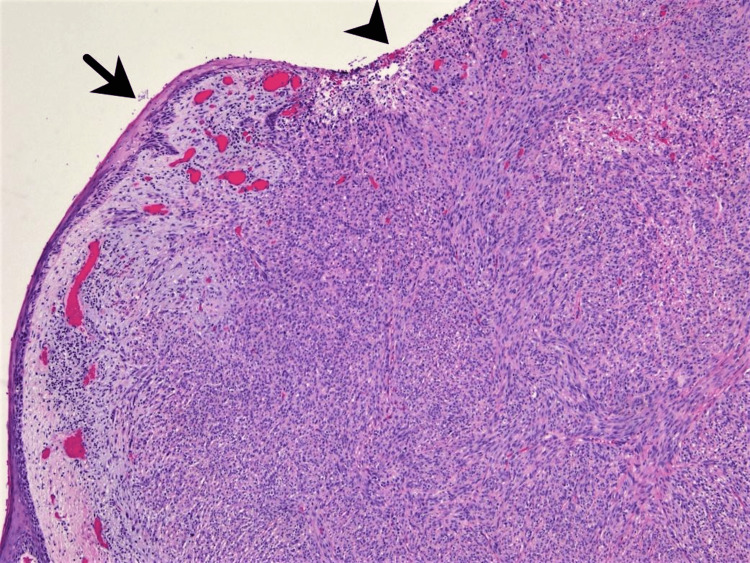
Hematoxylin & eosin (H&E) staining, 50x magnification Diffuse proliferation of spindled cells within the dermis is observed.  The epidermis, where intact (arrow), is attenuated; it is ulcerated in other areas (arrowhead).

**Figure 4:**
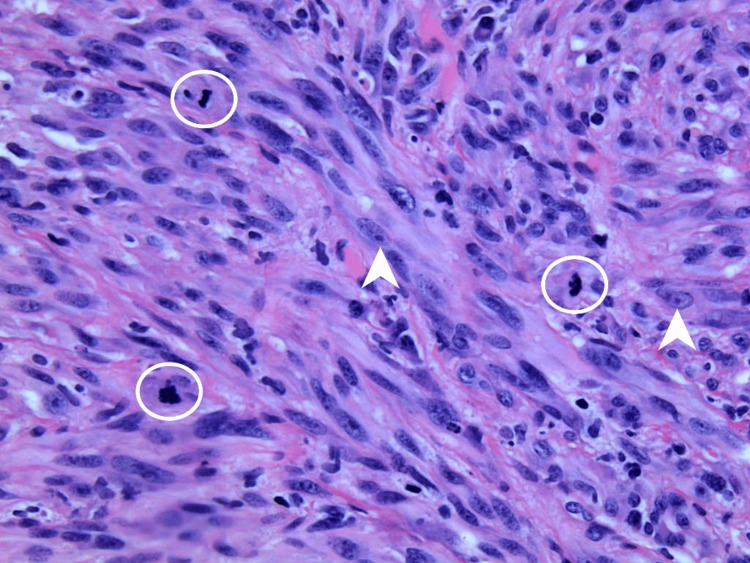
Hematoxylin & eosin (H&E) staining, 400x magnification Spindled cells exhibit marked pleomorphism characterized by nuclear enlargement, pleomorphism, prominent eosinophilic nucleoli (arrowheads), and increased mitotic activity (circles).

**Figure 5:**
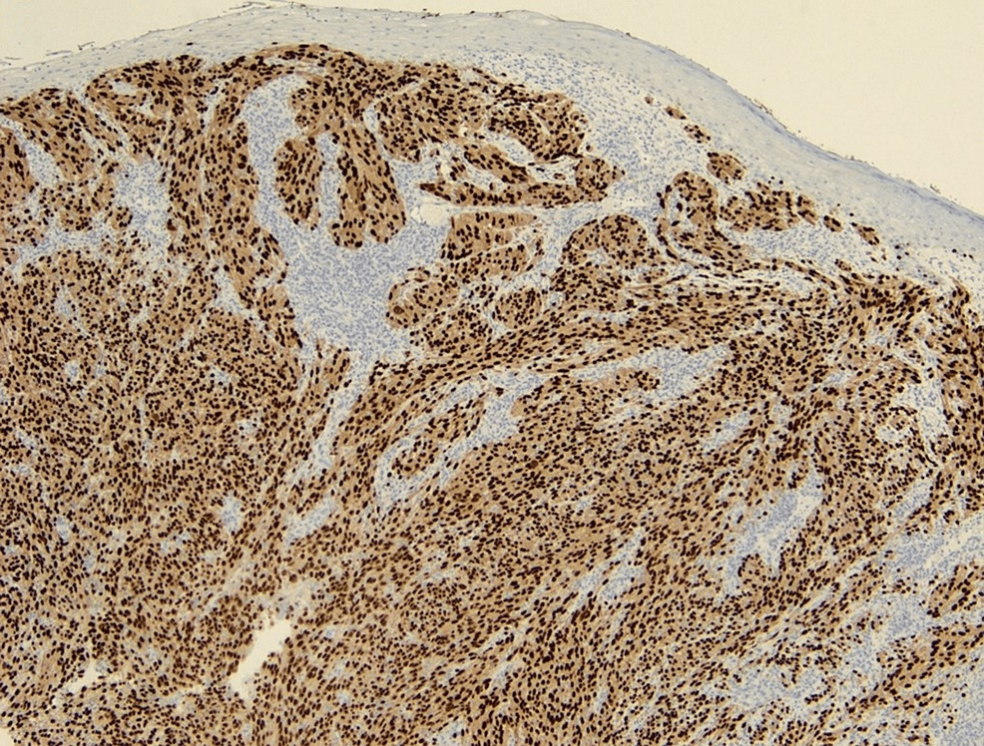
Histologic view of SOX10+ melanoma SOX10, 50x magnification. Lesional cells demonstrate nuclear immunoreactivity, consistent with a malignancy of melanocytic origin.

Pathological evaluation of the nasal mass showed a markedly pleomorphic spindled neoplasm, greater than 4.0 mm in thickness, with ulceration of the overlying epidermis (Figures 3-4). Lesional cells demonstrated diffuse nuclear immunoreactivity to SOX10, thus confirming invasive melanoma (Figure 5). Incorporating imaging studies, the melanoma was clinically staged as T4bNxM1d(1), stage IV disease. Her case was discussed with a multidisciplinary team, including the patient’s family, at a formal tumor board conference. Ultimately, the decision to surgically resect her nasal and scalp tumors was made for palliation. Further palliative measures included regional flap reconstruction of her nose and radiation therapy for the cerebellar lesion, which most likely represented metastasis from her melanoma.

**Figure 6:**
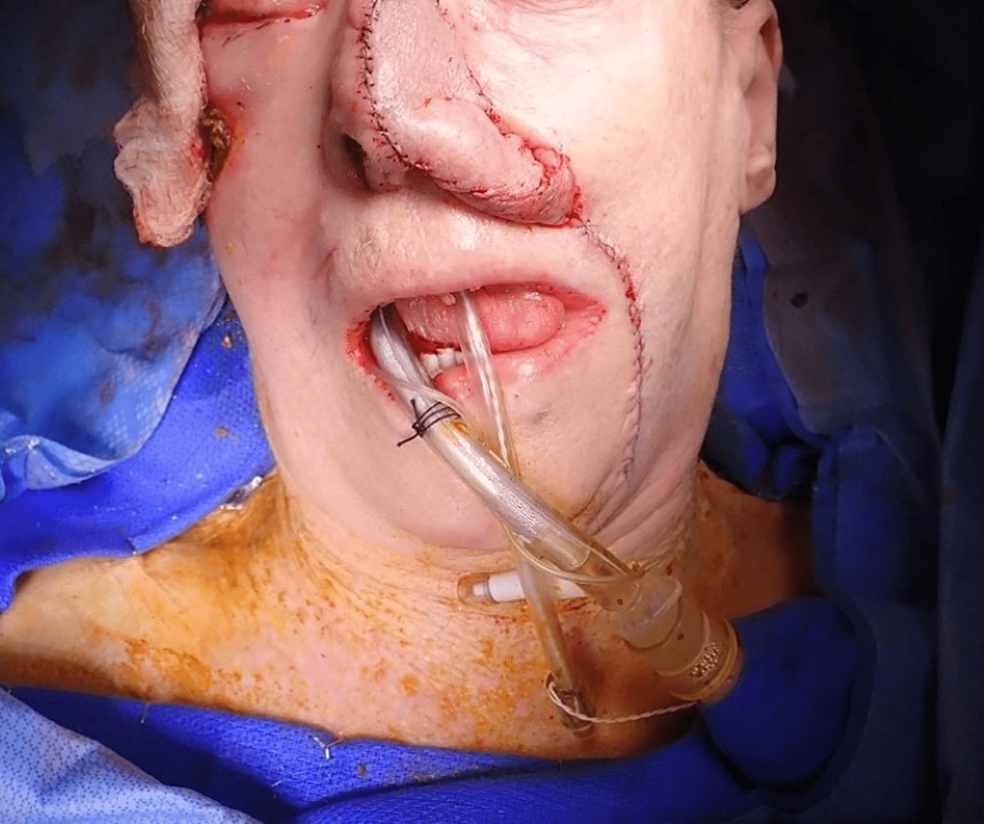
Melolabial flap creation Intra-operative photo after wide local excision of the scalp lesion and resection of the nasal melanoma with reconstruction, to include conchal and septal cartilage harvests and melolabial flap creation.

The patient consented to composite resection of the nasal melanoma, wide local excision of the scalp lesion, and subsequent reconstructive measures to include conchal and septal cartilage harvests, split-thickness skin grafting, and left inferior turbinate and melolabial flap transfers (Figure 6). Post-operatively, she received five sessions of radiation therapy for her cerebellar lesion.

During hospitalization, the melolabial flap appeared congested at the distal aspect and a hematoma was evacuated. After evacuation, the flap appearance remained stable. Vertigo worsened after each session of radiation therapy and persisted after completion. Similarly, the right-sided weakness also persisted despite daily attempts at physical therapy. Following her 56-day hospitalization, the patient was discharged to a skilled nursing facility given her inability to independently perform individual activities of daily living.

The patient was re-evaluated 12 days after discharge for division of the melolabial flap pedicle by the otolaryngology team. At that time, the inferior turbinate flap, septal and conchal cartilage grafts, and the distal melolabial flap appeared grossly necrotic, and there was a subsequent full-thickness defect of the lateral nasal wall. In the operating room, the vascularized pedicle of the melolabial flap was advanced into the defect for closure. Intraoperative biopsies of the skin surrounding the defect were sent for pathological evaluation and returned negative for residual melanoma. She returned to the skilled nursing facility upon discharge.

## Discussion

Cutaneous melanoma represents only 3% of all skin cancers diagnosed each year but disproportionately accounts for 65% of all deaths due to skin cancer [4]. The death rate is significantly lower than the incidence rate, likely because treatment of early melanomas is often curative [4]. Localized melanoma has a five-year survival rate of 99%, which decreases to 30% in the case of metastatic disease [2]. Treatment for metastatic melanoma can be divided into two broad categories: local and systemic [5]. Local treatment options include surgical resection of primary sites and metastatic lesions, stereotactic radiosurgery, and whole-brain radiation. Systemic treatment options include chemotherapy and immunotherapy [5]. Whenever possible, surgical removal with adequate margins is the first-line treatment of melanoma. Though it is mainly performed in patients with up to stage II melanoma, it is also often an option for stage III patients or even when the disease has already metastasized to other organs (stage IV). However, especially in some patients at stages II and patients at stages III and IV, surgery alone has limited curative potential. Thus, radiotherapy, chemotherapy, immunotherapy, or targeted therapy is often used as adjuvant treatments.

Historically, metastasectomy without adjunctive systemic treatment was viewed as counterintuitive [5]. This was largely because oncologic resection only conferred a localized solution, rather than addressing the underlying disease. In 2014, the first Multicenter Selective Lymphadenectomy Trial (MSLT-1) was an early large-scale study that demonstrated that resection in melanoma with distant metastasis conferred a significant survival advantage over systemic therapy alone. Median overall survival (OS) of 15.8 months in patients with complete metastasectomy compared with 6.9 months in patients with systemic therapy was observed [5]. More recently in 2017, a randomized clinical phase III trial called the Malignant Melanoma Active Immunotherapy Trial for Stage IV Disease (MMAIT-IV) demonstrated a 40% five-year OS rate for metastatic melanoma after metastasectomy; this was significantly higher than the five-year OS rate expected with systemic therapy alone [5]. 

Despite the benefit of surgical resection, it is evident that improving outcomes in melanoma hangs on two basic but important concepts: prevention and early detection. Prevention strategies should focus on promoting health literacy of the population, targeting modifiable risk factors, and raising awareness of the importance of sun protection measures. Additionally, maintaining close surveillance of individuals with high-risk lesions is also critical. Still, an early and accurate diagnosis of the disease is essential to improve outcomes.

The COVID-19 pandemic has had a wide-ranging, quantifiable global effect on social interaction, the economy, and healthcare [6]. Unfortunately, our patient’s case illustrates a less quantifiable impact of the pandemic: the psychological cost [7]. Her story highlights the importance of identifying and treating often curable medical conditions and malignancies before they progress to late-stage disease. Especially in the context of crisis, clinicians should strongly encourage patients to be active participants in their healthcare and support them in making medical or surgical decisions by discussing appropriate risks, benefits, and alternative treatments.

One way for healthcare providers to facilitate connecting with their patients is telehealth, which has become increasingly popular in the time of physical distancing during the pandemic [8]. Many medical practices began replacing in-person visits with telephonic and video-based office visits to mitigate viral transmission risk, but also to continue to provide medical care while allaying patients’ fears.

Relatively, the use of teledermatology has been less widespread, with clinicians citing difficulties incorporating the technology into their practices due to poor ability to visualize lesions, or the limited ability of patients to properly use the technology or properly display their lesions [9]. While far from ideal, standardizing the use of teledermatology could be of great utility-if not for formal on-the-spot diagnosis or treatment, then at least for triage. When combined with physical office visits, this has the potential to increase access to medical care, allowing for earlier patient education and treatment of suspicious skin lesions, and may alleviate patients’ fears, particularly during a medical crisis. We hope more patients begin or continue to utilize telehealth to avoid the mortality that simply waiting can confer to an otherwise treatable condition.

## Conclusions

The COVID-19 pandemic has revealed numerous watershed regions in the United States healthcare system that have contributed to worsened outcomes for many patients. Despite the scarcity of healthcare resources, physicians should seek to overcome barriers to accessing care, offer alternative solutions to in-person visits instead of cancellations, and strive for early diagnosis of curative conditions. Telehealth is an important, perhaps crucial, tool for extending physician outreach when diagnosing cutaneous melanoma during a healthcare crisis.
